# Critical Assessment of the Concepts and Misconceptions of the Cardiac Conduction System over the Last 100 Years: The Personal Quest of Robert H. Anderson

**DOI:** 10.3390/jcdd8010005

**Published:** 2021-01-19

**Authors:** Eduardo Back Sternick, Damián Sánchez-Quintana

**Affiliations:** 1 Electrophysiology Unit, Biocor Instituto, Nova Lima 34006083, Minas Gerais, Brazil; 2 Department of Anatomy and Cell Biology, Universidad de Extremadura, 06071 Badajoz, Spain; sanchezquintana55@gmail.com

**Keywords:** conduction system of the heart, atrio-his connection, atriofascicular pathway, fasciculo-ventricular pathway

## Abstract

Anatomical concepts regarding the conduction system of the heart have been a matter of debate since pioneering work done at the beginning of the 20th century. Robert H. Anderson was actively involved in this field for half a century. We aimed to investigate how his own concepts evolved over time. We have assessed anatomical concepts relating to the cardiac conduction system appearing since the key contributions made in the initial decade of the 20th century, analyzing them from the perspective of Robert H. Anderson, particularly focusing on the anatomical aspects of structures such as accessory atrioventricular pathways, including the so-called Mahaim-type fibers, connections between the atrioventricular node and the atrial myocardium, and so-called “specialized” internodal atrial tracts. To accomplish this task, we have taken as our starting point the initial concepts published in the first decade of the century, along with those subsequently reported up to 1976, and assessing them in the light of our most recently published works. The concepts put forward by Robert Anderson with regard to atrioventricular nodal bypass tracts, atrioventricular nodal inputs, decrementally conducting accessory pathways, and “tracts” for internodal atrial conduction, have remained consistent along the time frame of half a century.

## 1. Introduction

Robert H. Anderson was a West Point graduate, born in 1835, who served as a brigadier general in the army of the Confederate States during the American civil war. In spite of pursuing combat and potential victory on the battlefield, he subsequently played an important role with the efforts for reunification after the war. A different Robert H. Anderson, a British national, has dedicated most of his time to the research of the normal and congenitally malformed heart. Based initially at the Royal Brompton Hospital, supported by the British Heart Foundation, and known to his friends and colleagues as Bob, he has enjoyed a long, ongoing, and very productive career, with 1200 manuscripts and collaborative interaction with scientists all over the world. Like his namesake, who became famous at seeking reunification, Bob has worked together with other scientists and physicians to translate concepts of anatomy and embryology into relevant clinical and surgical information. Even his “enforced retirement” in 2007 did not diminish his impetus and drive to study. He is currently working at the Institute for Genetic Medicine, Newcastle University, Newcastle upon Tyne, and at Birmingham Children’s Hospital in the United Kingdom, but also collaborates extensively with colleagues based in the United States of America.

It has been an honor and joy for us to work with him in a number of projects, all designed to improve our understanding of cardiac arrhythmias and their anatomic–functional relationships. This current review is dedicated to his work in the field of cardiac arrhythmias, with emphasis on a particular set of sub-structures. In his early formative years, Professor Anderson spent a remarkably productive year at the University of Amsterdam, where he collaborated with pathologists and electrophysiologists, in particular with Giel Janse, Anton Becker, and Hein Wellens, with their studies at that time providing the basis for important subsequent contributions. In this review, we reappraise some of the concepts reported back in the 1970s in the light of current knowledge, mostly accumulated by himself.

## 2. Methods

We assessed anatomical concepts relating to the cardiac conduction system appearing since the key contributions made in the initial decade of the 20th century, analyzing them from the perspective of Robert H. Anderson, who has now been actively involved for half a century in understanding the anatomical aspects of structures such as accessory atrioventricular pathways, including the Mahaim-type fibers, connections between the atrioventricular node and the atrial myocardium, and so-called “specialized” internodal atrial tracts. To accomplish this task, we have taken as our starting point the initial concepts published in the first decade of the century [1,2,3,4], along with those subsequently reported up to 1976 [5,6,7], and assessing them in the light of our most recently published works.

In addition, we have taken advantage of a series of e-mails exchanged directly with Bob, aiming to unravel details not possible to discern from the published manuscripts themselves. References [5,6] are chapters from a book released in 1976 by Stenfert Kroese BV. The monograph had its genesis in a workshop on the cardiac conduction system held in the spring of 1975. It took place at the Department of Cardiology, Wilhelmina Gasthuis, Amsterdam, The Netherlands. The reference is important, since this was the home ground of one of the most outstanding electrocardiologists of the time, Dirk Dürrer, who made several important contributions to modern cardiac electrophysiology, and was the initial mentor of Robert H. Anderson. He created an amazing institute, where investigators in various disciplines cooperated in the study and treatment of cardiac disease. The book was dedicated to him by three of his outstanding pupils, Hein Wellens, KI Lie, and Michiel Janse. As we have already stated, Bob, early in his career, had spent a formative year training in the Netherlands. His special interest in the cardiac conduction system was nurtured at that point in time, thanks to the interactions with these luminaries of cardiac electrophysiology and pathology.

We will include in our reappraisal the concepts of atrioventricular nodal bypass tracts, as alleged to exist by James and Brechenmacher [8,9,10], the pathways described initially by Ivan Mahaim, and the controversies that still surround the presence of tracts within the atrial walls.

Concerning the technical aspects of examination of the conduction system, Bob has stated that his approach in the 1970s, and even now, was no different from that used by Tawara [1], namely the careful assessment of serial sectioned histological material. As Bob stated in one of his recent e-mails, “I have been fortunate throughout my career to have been able to work with people able to produce excellent histological material. The recent work done with Damian, who initially studied with me in London, investigating the myocardial architecture in tetralogy of Fallot, has served greatly to increase my knowledge. I also now have a much better understanding of the development of the heart. From the earliest times, however, I have always thought it was necessary to stress the differences between the species. Only now, however, am I in a position to emphasize the significant differences! The work we have done together over the past two to three years has served to bring everything together”.

## 3. Results

### 3.1. Atrioventricular Nodal Bypass Tracts

As Bob stated in 1976, the concept of nodal bypass tracts was introduced by James [8] on the basis of morphological studies carried out to provide an explanation of some abnormalities of atrioventricular conduction, which might then be distinguished electrophysiologically. He warned at that time that it was dangerous to directly extrapolate morphological findings to function. He pointed out the considerable variation to be found in the manner in which the transitional or nodal approach, cardiomyocytes, make contact with the compact node. He considered it preferable to return to the initial definition provided by Tawara [1], and to recognize the boundary between the compact node and the penetrating bundle as the point at which the atrial cardiomyocytes cease to make contact with the atrioventricular conduction axis. On this basis, the penetrating bundle can be considered to represent the final common pathway for normal atrioventricular conduction.

Using this definition, a morphological by-pass tract, without implying any functional significance, will be represented as any aggregation of atrial cardiomyocytes that make contact with the nodal-bundle axis distal to the point of origin of the penetrating bundle, in other words, inserting into the final common pathway distal to the compact atrioventricular node. Such a tract had allegedly been described by Brechenmacher and his colleagues [9,10] in a patient known to have exhibited electrophysiological abnormalities. In the experience of Bob, the last atrial cardiomyocytes to make contact with the compact node could be derived either from superficial overlay myocardium, or from the deep left side of the atrial septum. The anterior overlay cardiomyocytes were known to originate directly from the antero-inferior buttress of the atrial septum, and then to make contact with the compact node.

Bob had been unable to identify any aggregated cardiomyocytes coursing from the posteroinferior part of the atrial septum, as had allegedly been described by James. In Bob’s opinion, if the variations described by James were considered to be significant to nodal function, they should be interpreted as variations in nodal structure, and hence representing intranodal pathways, rather than considering them as by-pass tracts. If such pathways are, indeed, of functional significance, which is yet to be proven, then account must also be taken of the arrangement of the inferior extensions of the compact node. Variations in these pathways self-evidently can influence the route taken to the final common pathway. In this regard, we now know that the connection between the leftward inferior extension of the compact node and the left side of the atrial septum and the mitral vestibular myocardium is particularly noteworthy. As Scherf and Cohen [11] pointed out long since, the node is an interatrial structure, and not a right atrial structure.

**Figure 1:**
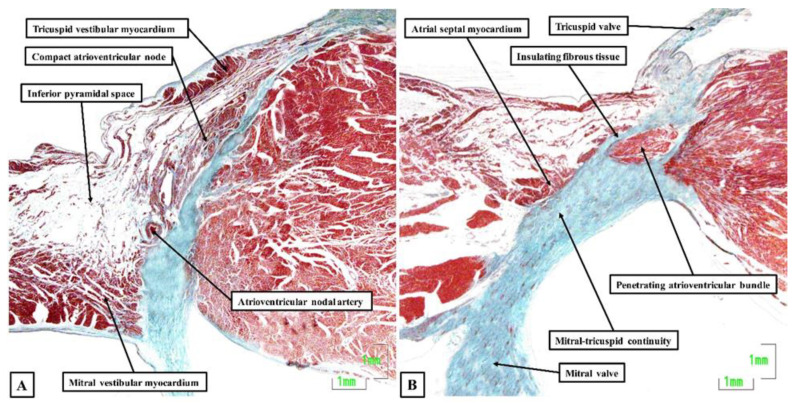
Atrioventricular node and penetrating bundle: The histological sections, stained using the trichrome technique, show the features of the atrioventricular node (**A**) and the insulation provided to produce the penetrating atrioventricular bundle (**B**). The sections were made using an adult human heart.

**Figure 2:**
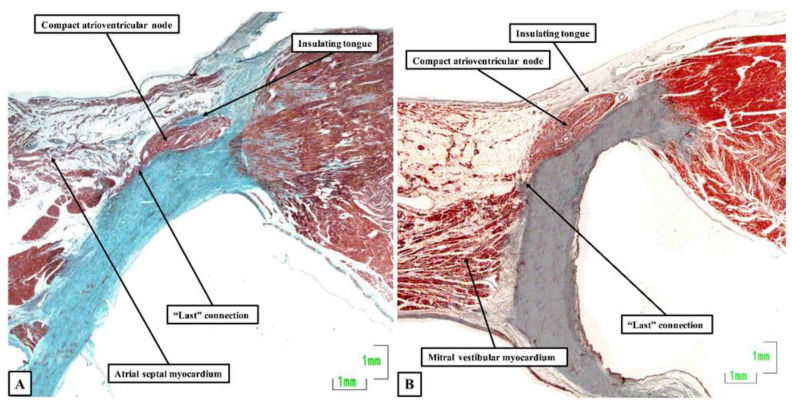
The compact node and the last connection: The sections, again stained using the trichrome technique, show the last connections to the atrioventricular node from the central part of the atrial septum (**A**) as opposed to the mitral vestibule (**B**). The section shown in Panel A is from the same series of sections as shown for the atrioventricular node and penetrating bundle in Figure 1. Both sections are taken from adult human hearts.

Now, in 2020, 54 years later, Bob and his colleagues, including ourselves, have reassessed the structure of the atrioventricular node and its atrial connections [12]. We achieved this by studying 20 human hearts, assessing serial histological sections that covered the entirety of the triangle of Koch and the cavotricuspid isthmus. We were able to determine the location of the atrioventricular conduction axis, and the connections between the specialized cardiomyocytes of the conduction axis and the adjacent working atrial cardiomyocytes. As expected, the atrioventricular node was found towards the apex of the triangle of Koch (Figure 1A), with insulation of the conduction axis by the fibrous components of the atrioventricular junction providing the criterion for distinction of the bundle of His (Figure 1B). We found marked variation in the inferior extensions of the node, the shape of the node, the presence or absence of a connecting bridge with the myocardium of the atrial septum, the presence of transitional cardiomyocytes, and in particular, the last connection between the working atrial myocardium and the conduction axis before it became the bundle of His. In the majority of datasets, the last input came from the central part of the atrial septum (Figure 2A), and was composed of working atrial cardiomyocytes. In some instances, nonetheless, it was found to arise from the left-side of the septum (Figure 2B). The observed variations in the extent of the inferior extensions, combined with the arrangement of the last connections between the atrial myocardium and the conduction axis prior to its insulation as the bundle of His, now provide compelling evidence to support the concept for atrioventricular nodal re-entry as previously advanced by Katritsis, working with Anton Becker [13].

### 3.2. The Pathways Described by Ivan Mahaim

#### 3.2.1. Fasciculo-Ventricular and Nodo-Ventricular Pathways

It was in the early 1940s that Ivan Mahaim, a Belgian cardiologist working in Switzerland, described the presence of “fines hautes connexions”, translated as delicate proximal connections, and also called paraspecific pathways, which connected the central part of the atrioventricular node and the penetrating bundle directly to the crest of the ventricular septum [14,15]. These entities were considered to be remnants of the embryonic anlagen of the conducting tissues. Indeed, such remnants are readily identifiable in infant, childhood, adolescent, and adult hearts, although in decreasing frequencies. Mahaim had concluded that these structures might serve as septal conduction pathways, providing alternative pathways to the bundle branches and their ramifications, but with a wide spectrum of variability in dimensions and locations. He attempted to demonstrate their functional role by showing experimentally that sequential cutting of these connections modified the surface electrocardiogram.

Bob, along with Janse and Becker, studied a fetal human heart in which multiple atrioventricular connections, as described by Mahaim, were shown to be present. During electrical stimulation of the atrium, however, atrioventricular conduction occurred only through the normal pathways for atrioventricular conduction [16]. In 1971, Wellens [17] reported the electrophysiologic findings in a young boy having paroxysmal tachycardia caused by an accessory pathway with decremental properties. That finding subsequently rekindled interest in the anatomic–functional relationship of the so-called Mahaim connections.

**Figure 3:**
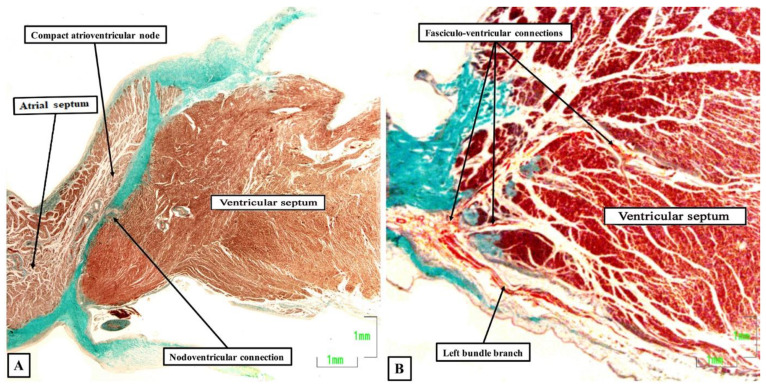
Nodo-ventricular and fasciculo-ventricular pathways: The sections are again stained using the trichrome technique. (**A**) shows a nodo-ventricular connection in an infant human heart, while (**B**) shows multiple fasciculo-ventricular connections in an adult human heart.

A few years thereafter, Anderson and his colleagues [5] suggested separating the pathways described by Mahaim into nodo-ventricular connections, which took their origin from the compact atrioventricular node (Figure 3A), and fasciculo-ventricular connections, which originated more distally from the atrioventricular conduction axis (Figure 3B).

**Figure 4:**
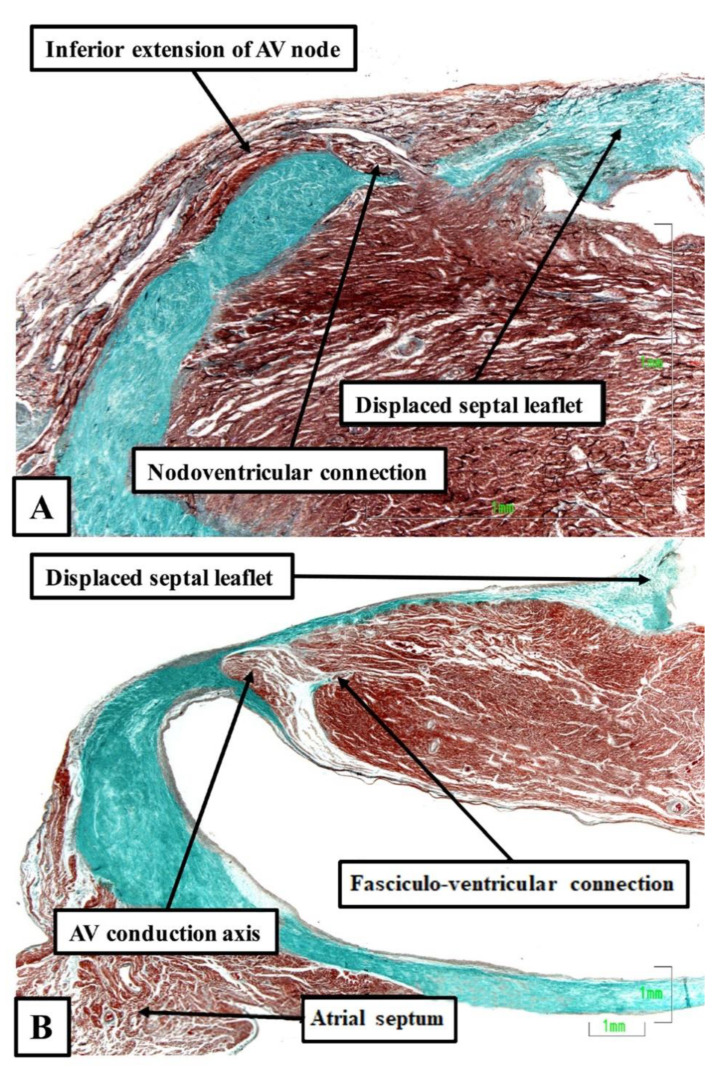
Nodo-ventricular and fasciculo-ventricular pathways: Both sections were taken from human hearts shown at autopsy to exhibit Ebstein’s malformation. (**A)** shows a direct nodoventricular connection between the inferior rightward extention of the atrioventricular (AV) node and the crest of the ventricular septum, while (**B)** shows a fasciculo-ventricular connection given off from the conduction axis prior to the origin of the right bundle branch. The sections were stained using the trichrome technique.

It was then Becker working with Gmeiner and colleagues, who were the first to document the anatomical existence of a functional nodo-ventricular pathway. This pathway was found in an eleven-year-old boy with a previous history of paroxysmal recurrent tachycardia, who had suffered a cardiac arrest while tobogganing. He was found in ventricular fibrillation, was resuscitated, but developed a persistent vegetative state for three years, and eventually died [18]. At autopsy, Becker removed the complete atrioventricular junction, including the left and right parietal zones as well as the septal junctional zone, for serial histological examination. On examination of the sections, he found a discrete tract of specialized cardiomyocytes, which extended obliquely from the base of the compact atrioventricular node, crossed the fibrous plane of atrioventricular insulation, and inserted into the crest of the muscular ventricular septum. Such nodo-ventricular connections have subsequently been shown to be ubiquitous in the setting of Ebstein’s malformation. Thus, Bob, again working with one of us and our clinical colleagues, [19] reported the findings in six autopsied hearts from patients known to have had Ebstein’s malformation. The lesion was fully developed in four, but was of a so-called “micro-Ebstein” form in two. All hearts had been studied subsequent to serial histological sectioning of the full extent of the atrioventricular conduction axis, along with limited sectioning of the right atrioventricular junction supporting the inferior and antero-superior leaftlets of the deformed tricuspid valve. In all of the hearts, overt nodo-ventricular connections were identified (Figure 4A). In two of the five, fasciculo-ventricular pathways were also present (Figure 4B).

Clinical correlations: fasciculo-ventricular pathways have not been reported to play an active role in re-entrant circuits, but they may be used as bystander structures. The nodo-ventricular pathway may be part of a re-entry circuit, and have a role as the retrograde limb or as the anterograde limb, giving rise to orthodromic or antidromic tachycardias, like in the case reported by Gmeiner and colleagues [18].

#### 3.2.2. Accessory Atrioventricular Node or Atriofascicular Pathway

Becker in 1978, working with Dürrer, Hein Wellens, and Bob [20] sought to establish whether the hearts also exhibited so-called “atriofascicular tracts”. In this investigation, which was focused on elucidating the pathways present in patients who had presented with Wolff-Parkinson-White syndrome, an accessory atrioventricular node was identified that gave rise to an insulated tract of specialized cardiomyocytes. The tract pierced the insulating pathways of the atrioventricular junction, and extended into the right ventricle, thus producing a second atrioventricular conduction system located on the lateral part of the tricuspid annulus. Due to the lack of specific structure–function correlation, their finding did not serve to challenge the prevailing concept that the structure responsible for so-called Mahaim conduction was, indeed, produced by nodo-ventricular connections, as described by Mahaim himself, and endorsed by the findings of Gmeiner and associates [18]. It was not until the early 1980s that it became clear that the pathway for the majority of instances of “Mahaim conduction” was instead mediated through accessory atrioventricular nodes. We are now well aware that these nodes themselves represent remnants of so-called atrioventricular ring tissue, shown to exist in the human heart by Bob, working along with Anton Becker and the late Michael Davies. We now describe these right sided and parietal pathways as being “atriofascicular”, recognizing that their ventricular termination can be with the right bundle branch [21,22].

The atriofascicular pathways, are usually the antegrade limb of the circuit of an antidromic tachycardia, while the retrograde limb is the atrioventricular conduction axis, or a second accessory atrioventricular pathway. It may also be a bystander structure when the patient has an additional accessory pathway or dual atrioventricular pathways and atrioventricular node re-entrant tachycardia.

### 3.3. Specialized Tracts for Internodal Atrial Conduction

**Figure 5:**
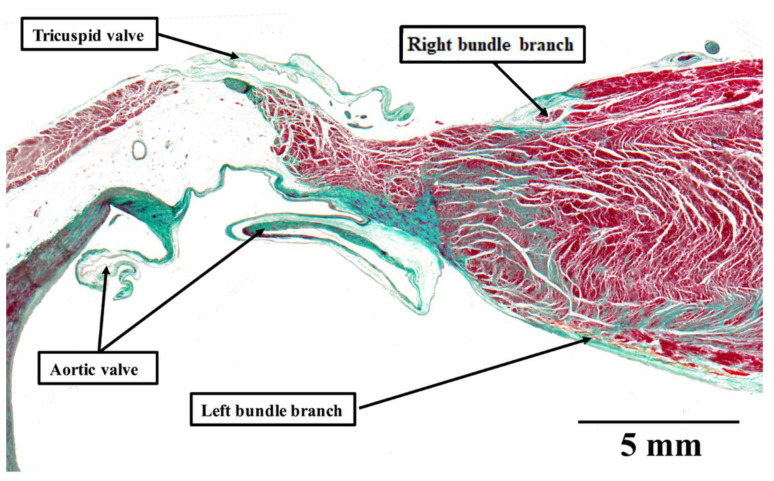
Mönckeberg and Aschoff’s criteria for a conducting tract: The histological section shows the crest of the ventricular septum in a human heart stained using the trichrome technique. The right bundle branch is insulated by a sheath of fibrous tissue, colored green. This makes it possible to distinguish the bundle from the adjacent myocardium, and follow the bundle in serial sections. Thus, the structure satisfies all of the criteria established by Mönckeberg and Aschoff [3,4] for recognition as a conducting tract.

**Figure 6:**
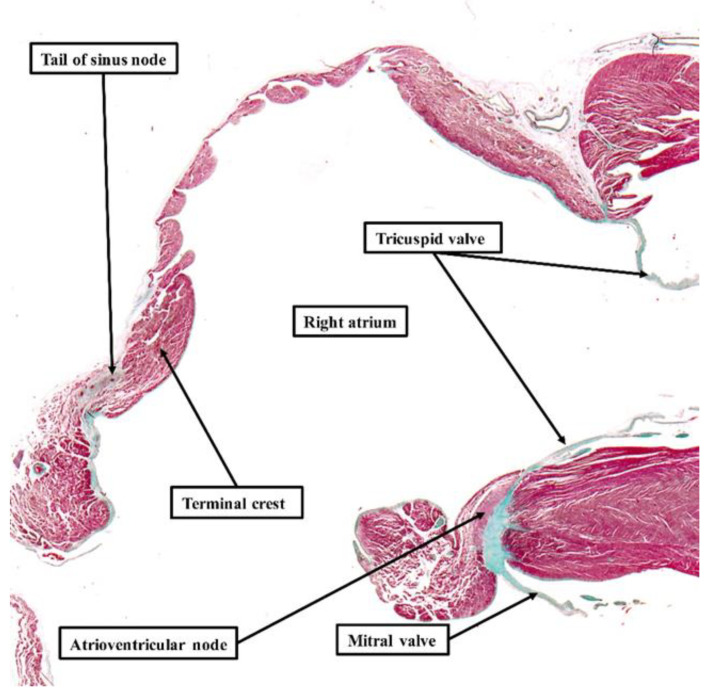
Absence of specialized tracts within atrial walls: The section is taken from a human of 35 weeks. It shows the entirety of the walls of the right atrium, with the section again stained using the trichrome technique. Even at the low power of magnification used to show the features of the right atrium, it is possible to recognize the tail of the sinus node and the atrioventricular node. The atrial walls, however, do not contain any insulated tracts comparable to the right bundle branch (see Figure 5). Only when assessing the entirety of the atrial myocardium in this fashion, using serial histological sections, is it justifiable to draw any conclusions regarding the presence or absence of “specialized” tracts responsible for conduction. When using the criteria established by Mönckeberg and Aschoff [3,4], which retain their validity for histological studies, examination of serial sections shows that there are no such tracts.

The pathways for conduction between the atrioventricular and sinus nodes [1,2,3], themselves newly described at the time, had been hotly debated by the end of the first decade of the 20th century. To resolve this conflict, a session of the meeting of the German Pathological Society held in 1910 at Erlangen was allocated to discuss the proposal made by Thorel that internodal conduction occurred through a specialized tract [20]. The session was attended by JG Mönckeberg, Ludwig Aschoff, Thorel himself, Walter Koch, James Mackenzie, and Thomas Lewis, among other luminaries. The key points emerging from the meeting were summarized by Aschoff and Mönckeberg [3,4]. They provided criteria for conduction tracts based on the identification of the right bundle branch, which they pointed out could be traced from section to section in histological material and was distinguishable from the adjacent myocardium on the basis of its histological appearance, and these features were insulated by a fibrous sheath (Figure 5). It was the last feature that was identified as the significant criterion for a conducting tract. All agreed that, on this basis, no such insulated tracts were to be found within the atrial walls separating the sinus and atrioventricular nodes. In 1910, therefore, the concept as advanced by Thorel was summarily dismissed. Throughout the subsequent decades of the 20th century, others made claims for the existence of the alleged pathways, but were always seemingly ignorant of the criteria proposed by Mönckeberg and Aschoff [3,4]. It was Thomas N. James, however, who subsequently brought the alleged pathways to the attention of cardiologists, such that they are currently depicted on the cover of “Heart Rhythm”. James, too, had also ignored the conclusion of the Erlangen meeting. Instead, he argued that the existence of such tracts had been proven on the basis of clinical findings, and hence his task was simply to illustrate their location [23]. In 2001, reviewing his anatomical investigations, he stressed that his original report on internodal pathways had been based on examinations of 69 human hearts [24]. Since then, he explained that he had examined more than 1100 additional human hearts, 89 dogs, 7 sperm whales, 9 cows, 7 horses, 12 rabbits, 6 cats, and a few rats, pigs, monkeys, nutria, chipamzees, and chickens. In each heart, he had studied two blocks of myocardium, which contained, on the one hand, the sinus node and on the other hand, the atrioventricular junctional tissues. He had examined each block on the basis of serial histological sectioning. There can be no question that he was a meticulous histologist, and the leading American cardiologist investigating the conduction system of the heart. It is self-evident, nonetheless, that his technique made it impossible to examine the entirety of the atrial walls so as to identify insulated pathways paralleling the insulation provided for by the right bundle branch (Figure 6).

His initial findings, published in 1963, furthermore, had already been challenged by the Dutch school represented by Dürrer, who had been unable to find electrophysiological evidence of such “specialized” tracts [25]. Becker and Anderson, who had examined the entirety of the atrial walls, pointed out that no anatomical evidence, when assessed on the criteria propounded in Erlangen [3,4], existed to support the presence of the tracts alleged to exist by James [23]. Janse, again working with Bob, subsequently emphasized that the walls of the right atrium were arranged around several “holes”, represented by the orifices of the caval veins, the coronary sinus, and the surrounds of the oval fossa. As they pointed out, when considering the anatomy delineated by these holes, four distinct pathways are present between the site of the sinus node and the atrioventricular junctional area [26]. These areas are rightly considered to be preferential pathways, but none of them contain “tracts” comparable to the insulated right bundle branch (Figure 5). As they emphasized, the mere presence of so-called “Purkinje” cells is not, by itself, a criterion for the existence of a tract specialized for conduction. Indeed, Spach and his colleagues [27] later showed that anisotropic conduction was more than enough to explain the preferential conduction through the atrial walls, which existed in the absence of insulated tracts. Thomas Naum James, nonetheless, along with other anatomists, continued to ignore the definition provided by Mönckeberg and Aschoff [3,4], supporting his hypothesis for the rest of his life.

## 4. Discussion

It is noteworthy that the views of Bob Anderson with regard to structures such as the inputs and extensions of the atrioventricular node, atrio-nodal, and atrio-His connections, the so-called “specialized” internodal tracts, and the pathways responsible for Mahaim conduction, have not changed over the period of half a century. On the contrary, Bob remembers that “when proposing the arrangements, we did not really have the evidence to back up the concepts. Much of the evidence needed came from the collaborative work with Damián, who is now able to “capture” the images as seen through the microscope, and make direct measurements [19,28,29,30]. He combines the gross photographs of the specimens with selected photographs of the serial sections, and prepares them in powerpoint presentations, sharing them with all of us. I can then choose selected images, crop or expand them to show the salient features. In this way, Damian has now shared datasets from over 70 adult hearts, 10 plus fetal hearts, a series of infant hearts with Ebstein’s malformation and controls, and examples of canine, porcine, and murine hearts. It is the ability to analyse this material “at a distance” on the basis of my prior knowledge that has enhanced my current understanding. It has been these spectacular and detailed datasets that have provided the much-needed evidence, particularly regarding the “atrioHisian” connections. I was dubious about these alleged pathways from the outset. And we have subsequently validated our skepticism concerning the publications of James! Our updated knowledge of development has also helped a lot”.

We are now in a position to assess the functional significance of these recent endorsements. With regard to the alleged atrioventricular nodal bypass tracts, it is certainly feasible that the last connection between the atrial cardiomyocytes and the atrioventricular node prior to its insulation as the bundle of His can provide potential pathways for the exit of proposed re-entry circuits for atrioventricular nodal reentrant tachycardia. It is likely that these last connections were previously misinterpreted as “atrio-Hisian connections” [9,10]. In reality, we now know that these connections are ubiquitous. As we showed in our collaborative study, their connection with the compact node just prior to its insulation as the bundle of His involves a minimal, if any, layer of interposing transitional cardiomyocytes. This paucity of nodal tissue can explain the absence of decremental conduction, as well as the lack of response to adenosine in the retrogradely conducting “fast” nodal pathway.

The entities were described initially by Mahaim, Bob, and his colleagues in 1975 and were separated into fasciculo-ventricular and nodo-ventricular connections [5]. This distinction still holds good. In the study revealing the connections of the atrioventricular node, we found one fasciculo-ventricular connection in the 20 hearts examined [12]. In an ongoing more extensive study, however, using datasets that contain more extensive parts of the ventricular components of the atrioventricular axis, and are as yet unpublished, we discovered fasciculo-ventricular pathways in one-third of the datasets (Figure 6). These findings are consistent with a survey of electrocardiographic screenings recently carried out in asymptomatic Japanese first- and seventh-grade children. This revealed two-thirds of 30 children with pre-excitation to have a fasciculo-ventricular pathway as the anatomic substrate [31]. Taken together with our histological findings, this raises the possibility that fasciculo-ventricular pathways may be the most common substrate for ventricular pre-excitation.

The concept that decrementally conducting accessory pathways might be related to the remnants of the atrioventricular ring, already reported in 1974, was revisited in 2009. The more detailed investigation included the reporting of the retroaortic node [32]. Although not associated with the variants of pre-excitation, Bob and his colleagues, again including one of us, proposed it to be a potential substrate for a variety of adenosine-sensitive atrial tachycardias [33].

Bob has also commented with regard to what is likely to be the very first histologically recognized atriofascicular pathway, which they had identified in 1978 [20]: “With regard to the accessory atrioventricular node, we had already discovered the atrioventricular ring tissues, and had shown that remnants could be found in all normal human hearts [34]. I had shown the presence of the “rings” in experimental animals using staining for choline esterase, again employing serial sections. Hein Wellens had studied the ECGs of all the patients, but the individual with the accessory node had multiple pathways, along with Ebstein’s malformation. The heart was huge, and it was an amazing undertaking simply to analyse the entirety of the atrioventricular junctions. We had to cut serial sections through 20 or more individual blocks, and then try to put the pieces back like a jigsaw puzzle! Becker’s histology technician was the real star!”.

If we conclude by returning to the so-called “specialized” atrial pathways, in Bob’s own words: “This topic was debated at length in 1910, and perfect definitions for insulated tracts were provided by Aschoff and Mönckeberg. Those definitions retain their validity. It is a simple fact that there are no insulated pathways extending between the sinus and atrioventricular nodes. The preferential conduction is well explained on the basis of the manner of aggregation of the individual cardiomyocytes. The claims for “specialization” on the basis of immunocytochemistry have never provided evidence of conduction ahead of that adjacent to the alleged “pathways”. With regard to Bachmann’s bundle, if you read his original account you will find that he explains that is the alignment of the cardiomyocytes in the anterior interatrial wall that is responsible for the preferential conduction! He was well ahead of his time!!”

## 5. Conclusions

Thus, we are able to conclude that the concepts put forward by Bob with regard to atrioventricular nodal bypass tracts, atrioventricular nodal inputs, decrementally conducting accessory pathways, and “tracts” for internodal atrial conduction, have remained consistent along the time frame of half a century. This is very unusual, given that the addition of new technologic tools, combined with critical reappraisal by other investigators, very often allows for a new perspective on a myriad of issues. There can be no question that his concepts have passed the test of time.
